# All-Atom Molecular Dynamics Simulations on a Single Chain of PET and PEV Polymers

**DOI:** 10.3390/polym14061161

**Published:** 2022-03-14

**Authors:** Mattanun Sangkhawasi, Tawun Remsungnen, Alisa S. Vangnai, Rungtiva P. Poo-arporn, Thanyada Rungrotmongkol

**Affiliations:** 1 Program in Biotechnology, Faculty of Science, Chulalongkorn University, Bangkok 10330, Thailand; mattajung@gmail.com; 2 Faculty of Interdisciplinary Studies, Nong Khai Campus, Khon Kaen University, Nong Khai 43000, Thailand; 3 Center of Excellence in Biocatalyst and Sustainable Biotechnology, Faculty of Science, Chulalongkorn University, Bangkok 10330, Thailand; alisa.v@chula.ac.th; 4 Department of Biochemistry, Faculty of Science, Chulalongkorn University, Bangkok 10330, Thailand; 5 Biological Engineering Program, Faculty of Engineering, King Mongkut’s University of Technology Thonburi, Bangkok 10140, Thailand; rungtiva.pal@kmutt.ac.th; 6 Program in Bioinformatics and Computational Biology, Graduate School, Chulalongkorn University, Bangkok 10330, Thailand

**Keywords:** polyethylene terephthalate, polyethylene vanillate, bio-based polymer, glass transition temperature, molecular dynamics simulation

## Abstract

Polyethylene vanillic (PEV), a bio-based material, has mechanical and thermal properties similar to polyethylene terephthalate (PET), the most common polymer used in industries. The present study aimed to investigate and compare their structural dynamics and physical data using a computational approach. The simple model of a single-chain polymer containing 100 repeating units was performed by all-atom molecular dynamics (MD) simulations with refined OPLS–AA force field parameters. As a result, the flexibility of the PEV structure was greater than that of PET. PET and PEV polymers had the predicted glass transition temperature *T_g_* values of approximately 345 K and 353 K, respectively. PEV showed a slightly higher *T_g_* than PET, consistent with current experimental evidence.

## 1. Introduction

An accumulation of plastic waste in the environment has received much attention worldwide [1,2] since it contributes to the ultimate causes of death of wildlife and marine animals. Macro-sized plastics such as pellets or shopping bags can resemble food for some hungry animals and cause them to die from digestive problems after ingestion [3]. The micro-sized plastics can be adsorbed on the surface or ingested by a range of aquatic organisms, including plankton, fish, bivalves, and even seabird [4]. Since some of these organisms are consumed by humans as food, these adsorbed particles possibly affect human health [5].

The massively used packaging materials are petrochemical-based plastics, e.g., polyethylene (PE), polypropylene (PP), and polyethylene terephthalate (PET). Since they are not recyclable and biodegradable, their uses become restricted. Instead, bio-based polymers have been successfully applied for food packaging [6,7]. These bioplastics can reduce plastic waste by replacing conventional petroleum-based monomers with biodegradable monomers. However, the production costs of various biomasses still limit the applications of these bio-based plastics in recent years.

**Figure 1:**
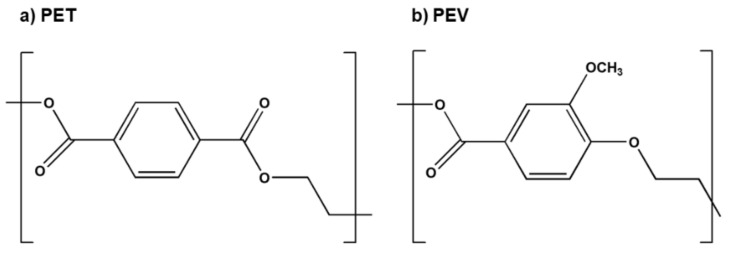
Chemical structure of (**a**) polyethylene terephthalate (PET) and (**b**) polyethylene vanillate (PEV).

Polyethylene vanillate (PEV), a bio-based polymer prepared from vanillic acid (Figure 1), is considered a potential PET alternative. Both polymers share similar mechanical and thermal properties, such as melting, glass transition temperature (*T_g_*), and cold-crystallization temperatures [8,9,10,11,12,13,14]. Moreover, thermal degradation of PEV also shared a common mechanism to woody and biomass [11], suggesting that PEV might be a biodegradable polymer to overcome the plastic waste problem and replace PET in the future.

The *T_g_* is an essential property in considering the application of polymers. It is related to when the polymer changes from a glassy state to a rubbery state. At the *T_g_*, the polymer suddenly increases its physical properties such as specific heat capacity, thermal expansivity, motions of molecule chains, and other parameters. There are several experimental methods for examining the *T_g_* of polymers, such as differential scanning calorimetry (DSC), ellipsometry, dynamic mechanical test (DMA), and infrared spectroscopy (FTIR). Molecular dynamics (MD) simulations can provide the polymer behaviors and thermal properties at the molecular level, e.g., density, specific volume, and *T_g_* [15,16]. The succession of MD simulations is strongly dependent on the choice of molecular force fields [17,18,19,20,21]. If possible, the all-atom (AA) model is the most suitable choice to obtain structures and conformations of molecular systems due to their ability to capture hydrogen-bond, Coulomb, and Lennard–Jones interactions [21]. As all of the atoms and interactions are presented and included in calculations, the MD simulations with the AA model are limited for systems with many particles and need a long simulation time such as hundreds of nanoseconds. The united-atom (UA) [19,22] and coarse-grained (CG) [18,20] force field models are developed to reduce the molecular degree of freedoms, i.e., site–site interactions. However, the parameters of the interaction of the CG model need to be verified by available experiments or simulation data based on the AA force field model [23]. There are some MD studies on PET using the AA model [24], UA model [19,22], and CG model [20,23,25,26,27,28], but the MD simulation of PEV with any force field models has not appeared yet. Thus, in this study, we aimed to investigate and compare the structural and physical properties of PET and PEV using the MD simulations with AA model on a single-chain polymer containing the 100 repeating units of ethylene terephthalate and ethylene vanillate, respectively. We modified and tested the general force field OPLS–AA for PET and PEV polymer simulations using *T_g_* as the primary validation index. The resulting structural, energetical, and thermal properties can further be used to optimize UA or GC parameters for PEV.

## 2. Materials and Methods

### 2.1. Molecular Structures and Their Force Fields

**Figure 2:**
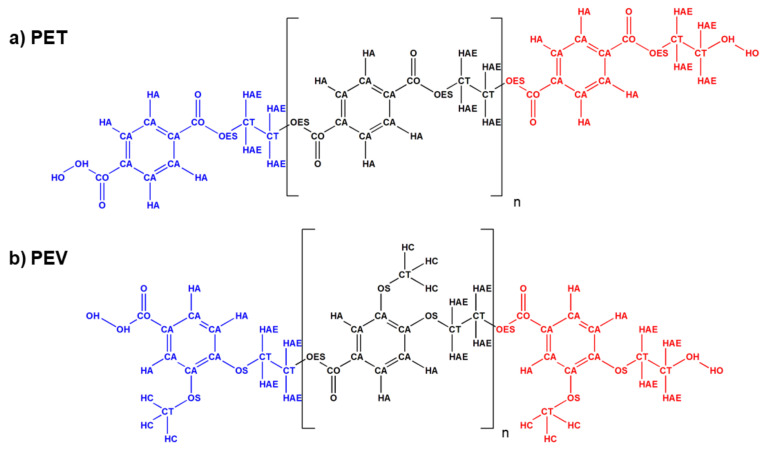
The atom types assigned for the two single-chain polymers: (**a**) PET and (**b**) PEV. The head, middle and tail of structures were represented in blue, black and red, respectively.

The molecular structures of trimeric PET and PEV were individually optimized at the B3LYP/6-31 + G(d) level of theory using the GAUSSIAN09 package, Revision B.01 [17]. The corresponding partial atomic charges were obtained using Merz–Kollman (MK) method [18]. Each single-chain polymer was divided into three parts, i.e., head, body, and tail, as shown in Figure 2. The partial charges were slightly rounded and refined to keep zero of the total charge of a polymer chain. The all-atom parameters of general force field OPLS–AA [19,20] were applied for intramolecular and van der Waal interactions. The DL_FIELD 4.1 [21] was used for force field assignment. All applied atomic partial charges and corresponding atom types are summarized in Tables S1–S4 of the Supporting Materials.

### 2.2. MD Simulations

The two single-chain polymers, PET and PEV, were conducted by all-atom MD simulations in periodic boundary conditions, with the NPT ensemble using the DL_POLY package version 4.0 [22]. The simulation time step was set to 1.0 fs using the leapfrog integration algorithm. The Berendsen thermostat and barostat with a relaxation time of 1.0 ps were applied. Nonbonded interactions were considered using the short-range cutoff of 12 Å with shifted Coulombic potential correction. The starting configuration unit cell contained a long linear chain of polymer in a big enough cubic box, i.e., the simulations of one chain of 100 repeating units in the cubic box lengths of 1200 Å and 1500 Å for PET and PEV, respectively. The pre-equilibrium at high temperature (600 K) and high pressure (250 atm) was performed for 2.0 ns to reduce the box size to its equilibrium density. Then, the simulation at 600 K with 1.0 atm was applied for 2.0 ns for system equilibration, and then a production run was carried out until 50 ns. The last configuration from the 2.0 ns simulation at 600 K was used as the starting configuration of lower temperatures step by step until 100 K. The simulation at each temperature comprised 2 ns of equilibration and then 48 ns of production. Each subsequent simulation was started from the 2 ns point of the adjacent equilibration run. Since each successive run was equilibrated from the last configuration of the higher temperature without hard scaling, it may take time to find the new equilibrium at the assigned temperature (the energetic data of both polymers may suddenly drop in the first 10–15 ns). The MD trajectories were saved every 0.25 ps for analysis.

### 2.3. Structural Characterization

The snapshots were extracted from the last 2 ns MD simulations for structural analysis in terms of the total and site–site radial distribution functions (RDFs) and dihedral angle distributions of the folded polymer. All RDFs were evaluated by the visual molecular dynamics (VMD) program version 1.9.3 [23]. All atoms of PET and PEV were included with a cutoff at 10 Å for the total RDF calculation, while the site–site RDFs described the molecular structure and chain folding from the CA atoms of the polymer benzene ring to the CT ethylene carbons and ester components, i.e., CO, O, and OES atoms.

### 2.4. Prediction of Glass Transition Temperature

The final specific volumes of simulation boxes were plotted versus the temperatures from 100 K to 600 K. The *T_g_* of the polymer was predicted at the intersection between the two trending lines at low temperatures of 100–300 K and high temperatures of 400–600 K. It is worth noting that the prediction of *T_g_* by monitoring specific volume or density of system per temperature was successfully applied in several polymers [15,16,24,25]

## 3. Results and Discussion

### 3.1. Folding of a Single-Chain Polymer

**Figure 3:**
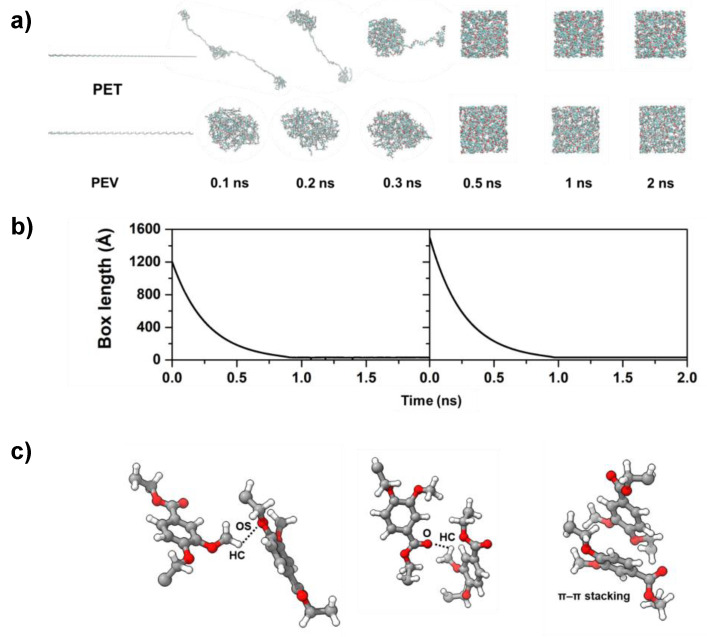
(**a**) The folding conformations of single-chain polymers PET and PEV upon simulation time at 600 K and 250 atm, where the plot of box lengths is shown in (**b**); (**c**) the possible hydrogen bonds formed between the methoxy group on the aromatic ring of a PEV unit and the ether and carbonyl oxygens of the neighboring units. Parallel-displaced π–π stacking between the aromatic rings is also presented.

The long linear chain of PET and PEV polymers was built from optimized repeating units and placed into the large and long enough simulation box length. All-atom MD simulations of such single-chain polymers were performed using an NPT ensemble at 600 K. The change in box length and polymer folding upon the simulation time was plotted and is depicted in Figure 3. It can be seen that a long polymer chain was folded by its intramolecular and intermolecular forces derived from the applied OPLS–AA force field. The use of high pressure at 250 atm in the pre-equilibrium state increased the rates of polymer folding and box length reduction. The simulation box length was dramatically decreased within 1.0 ns, from 1200 Å to 30.2 Å for PET, and from 1600 Å to 30.7 Å for PEV, close to the box length of 29.0 Å derived from an experiment density of 1.30 g/cm^3^ of PET [26]. By structural comparison upon folding process relative to PET, a single-chain PEV was folded more rapidly. This was because one ester group of PET was substituted by ether in PEV, and the methoxy group on the aromatic ring of a PEV unit interacted with other units and held them close together through hydrogen bonding with the ether and ester oxygens. Moreover, the aromatic rings of some monomers were orientated in parallel-displaced π–π stacking. On the other hand, the higher steric effect caused by this methoxy group may affect a slightly larger simulation box length by about 0.5 Å. The 2 ns configuration was used as starting structure of simulation at 600 K and 1.0 atm, and then the simulations at 1.0 atm were carried out at lower temperatures step by step with 100 K interval to 100 K. Each subsequent simulation was started from the 2 ns point of adjacent equilibration run.

**Figure 4:**
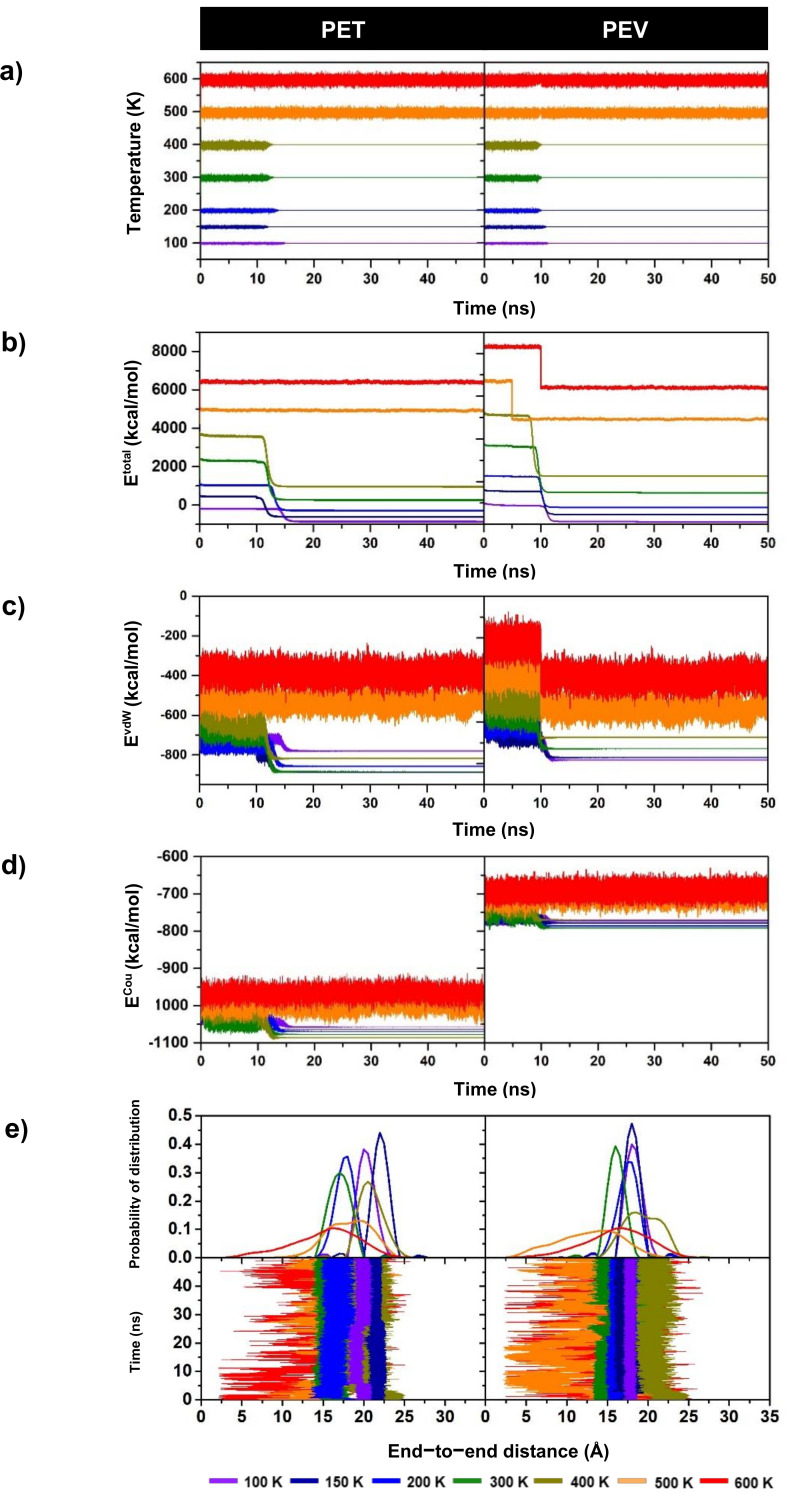
The plots of (**a**) temperature, (**b**) total energy (E^tot^), (**c**) Van der Waals energy (E^vdW^), (**d**) Coulombic energy (E^Cou^), and (**e**) end-to-end distance and its probability distribution for single-chain polymers PET and PEV along the simulation time at temperatures 100–600 K.

The MD simulations of PET and PEV at six different temperatures from 100 K to 600 K were investigated in terms of the temperature, total energy and its nonbonded components, and the distance between end to end of a single-chain polymer. All results plotted versus simulation time are given in Figure 4. Figure 4a–d show no significant change in temperature and energetics data after 20 ns at all temperatures, suggesting that all simulations had reached equilibrium. The end-to-end chain distance (Figure 4e) also supported the equilibrium of the systems; however, at high temperatures 500–600 K, this distance likely fluctuated in particular for the more flexible polymer PEV. The results of PET and PEV simulations during the last 2 ns were further analyzed and are discussed in terms of total and site–site radial distribution functions, dihedral angle distributions, and *T_g_* prediction in the following sections.

### 3.2. Structural Properties of Folded Polymer

#### 3.2.1. The Total Radial Distribution Functions

**Figure 5:**
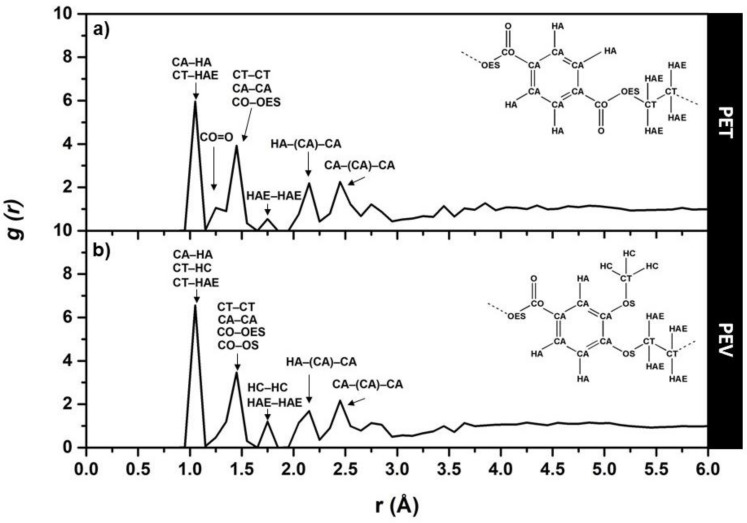
Total RDFs, g(r), for simulations at 300 K and 1 atm of (**a**) PET and (**b**) PEV polymers. Generally, the intramolecular peaks of the monomer unit are located within 4.50 Å. The atoms CA, CO, CT, O, OES, OS, HA, and HAE represent the aromatic carbon, carbonyl carbon, ethylene carbon, carbonyl oxygen, ester oxygen, ether oxygen, aromatic hydrogen, and ethylene hydrogen, respectively.

The total radial distribution functions (RDFs) plotted in Figure 5 provided information about the molecular structure features of PET and PEV. Here, only the simulated results at 300 K are discussed. In general, all peaks located within 4.50 Å were related to intramolecular site–site distances of the monomer unit. For PET, the first peak at about 1.1 Å attributed to CA–HA, and CT–HAE bonds, while the second peak at about 1.20–1.60 Å contributed to CO–OES single bond, ethylene CT–CT bond, aromatic CA–CA bond, CT–OS bond, and carbonyl CO–O double bond (subpeak at about 1.25 Å). The peaks that appeared after 1.60 Å corresponded to the distance between atoms with two bonds apart, such as a minor third peak at about 1.75 Å corresponding to the HAE–HAE lengths in the ethylene group. The following two peaks at about 2.15 Å and 2.45 Å represented the HA–CA lengths in HA–(CA)–CA and CA–CA lengths in CA–(CA)–CA of terephthalate ring, respectively. The total RDF results of PET agreed well with those of the previous study using polymer-consistent force field (PCFF) and condensed-phase optimized molecular potentials of atomistic simulation studies (COMPASS) [27]. In the case of PEV containing only one ester group, the subpeak at about 1.25 Å of the second peak corresponding to the CO–O double bond in the ester group was less pronounced than in PET. Instead, the third peak at about 1.75 Å was more prominent according to the HC–HC distances in the lateral methoxy group. All other peaks appeared in the same manner as those RDFs of PET. The obtained total RDFs showed the preservation of monomer units’ molecular structures and reasonably provided both similarities and differences between these two polymers. The OPLS–AA force field used in the present study is thus suitable for applying in PET and PEV simulations.

#### 3.2.2. The Site–Site Radial Distribution Functions

**Figure 6:**
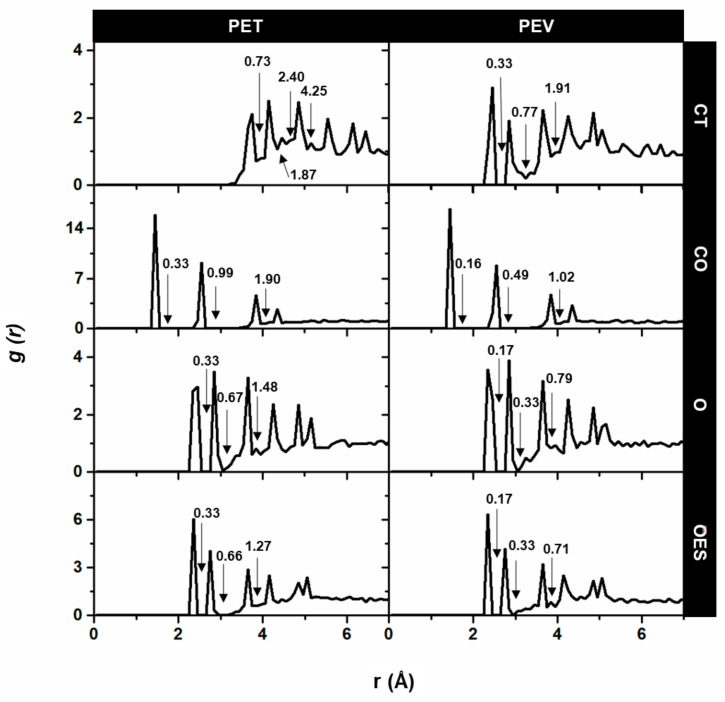
The site–site RDFs, g(r), centered on the CA atom of the polymer benzene ring to the CT ethylene carbons and the ester components CO, O, and OES in PET and PEV, where arrows show the coordination numbers up to the minimum.

The sites–site RDFs centered on CA aromatic carbons to the CT ethylene carbons, CO carbonyl carbons, O carbonyl oxygens, and OES ester oxygens of PET and PEV are plotted in Figure 6. As expected from their structures, the benzene ring of PEV was closer to the CT ethylene carbons, that is, the first sharp peak centered at around 2.35 Å, with the integral number up to the first minimum (coordination number n(r)) of 0.33, while that of PET was found to be at 3.45 Å, with higher n(r) of 0.73. Accordingly, the other sharp peaks were observed up to 5.05 Å and 6.15 Å in PEV and PET. Both polymers shared a very similar pattern in the rest of RDFs (CO, O, and OES) but differed in n(r). This was due to the morphology of PET containing two ester groups in contrast to PEV having only one group.

### 3.3. Dihedral Angle Distributions

**Figure 7:**
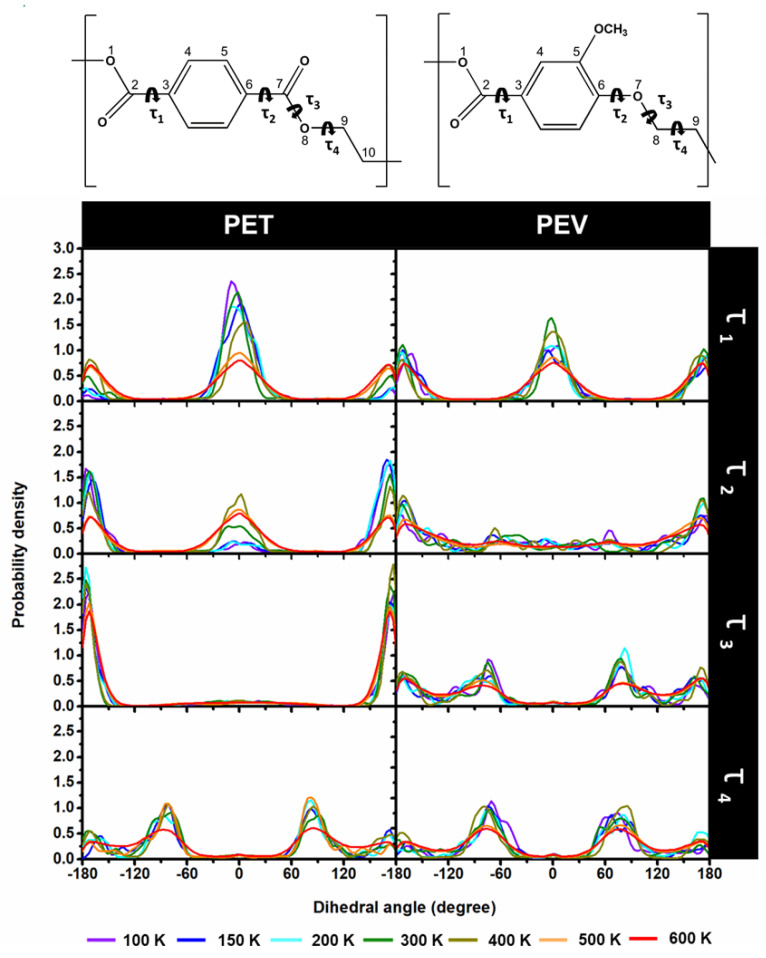
Distributions of the dihedral angles τ_1_ (1234), τ_2_ (5678), τ_3_ (6789) and τ_4_ (78910) of PET and PEV at different temperatures from 100 K to 600 K. The curved arrow was represents the dihedral angles.

To evaluate the polymer conformation of PET and PEV, the distributions of four dihedral angles τ_1_–τ_4_ at different temperatures were plotted and compared, the results of which are shown in Figure 7. By considering the higher peak of torsions, the τ_1_ (O1–C2–C3–C4) and τ_2_ (C5–C6–C7–O8) in PET were found at around 0° and −173/173° at low temperatures, i.e., the two esters orientated in the opposite direction. The probability of τ_3_ (C6–C7–O8–C9) was preferentially at –177/177° to establish a stable resonance form, while the τ_4_ (C7–O8–C9–C10) related to the rotatable bond showed higher flexibility. These τ_1_, τ_2_, and τ_3_ of PET representing the intramolecular orientation of the monomers agreed well with previous research [27]. Changing one ester group to ether in PEV led to a reduced distribution of τ_1_ (O1–C2–C3–C4) and τ_2_ (C5–C6–O7–C8) angles at almost the same angles as PET in particular τ_2_. This is due to a resonance structure with a lower number of total bonds in PEV. The torsions of the two single bonds τ_3_ (C6–C7–O8–C9) and τ_4_ (C7–O8–C9–C1) were somewhat varied in PEV. All structural information suggested a more rigid structure in PET, as mentioned above. In addition, an increase in temperatures could gradually raise the structural flexibility of single-chain polymers.

### 3.4. Glass Transition Temperatures

**Figure 8:**
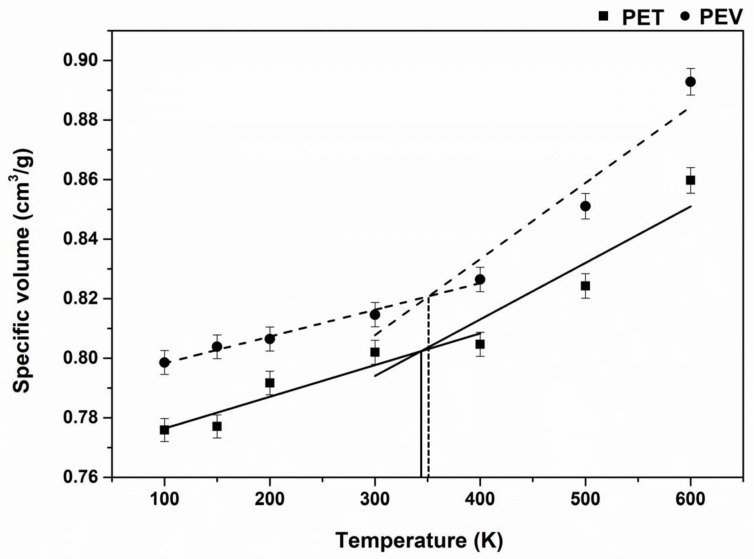
Temperature dependence of the specific volume for PET (■, solid line) and PEV (●, dashed line). The intersection of the two trended lines defined the glass transition temperature (*T_g_*) of the polymer.

The glass transition temperature, *T_g_*, is the essential key index to determine the polymer types of uses and modify physical properties. The *T_g_* prediction by MD simulation was reported in several polymers such as polyisobutylene [15], polystyrene [15], polyethylene [16], isomeric polyamide [24], and polyhydroxyalkanoate [25]. The specific volumes obtained from the simulation boxes at various temperatures from 100 K to 600 K for the two considered polymers were plotted and are shown in Figure 8. It can be seen that the increasing rate of specific volumes was not constant over all the temperatures. The slope of the trending line at high temperatures 400–600 K was greater than that of low temperatures 100–300 K. The *T_g_* values of the focused polymers estimated from the intersection of these two trending lines in Figure 8 were compared with the previous MD studies and experiments in Table 1. Our obtained *T_g_* value for PET of 345 K was very close to the previous prediction of 342 K [11] but somewhat lower than the available experimental values by 5–8 K [11,27,28]. Although the *T_g_* of PEV was first reported here at 353 K higher than that of PET in line with the experimental data (356 K) [10,11], it was 6–7 K more elevated than that of the others [10,29]. In addition, the density of PET was in a range of 1.30–1.40 g/cm^3^ [26,30]. As the reciprocal of specific volumes, the predicted densities of PET and PEV at 300 K and 1 atm were of c.a. 1.26 g/cm^3^ and 1.23 g/cm^3^, respectively.

## 4. Conclusions

PEV, a bio-based polymer, is considered as an alternative polymer to replace or blend with PET. Herein, we aimed to study the structural dynamics and thermodynamic properties such as *T_g_* of the polymers PET and PEV using all-atom MD simulations with the OPLS–AA force fields. The structural characterization from the plots of total RDFs and site–site RDFs mostly shared a similar pattern due to the preservation of monomer units’ molecular structures in the two polymers. Several different points are derived from the additional methoxyl group and the introduction of the ester group in PEV. In addition to the parallel-displaced π–π stacking between the aromatic rings, the methoxy group could interact with the ether and ester groups of the neighboring units via hydrogen bonding. The distributions of dihedral angles indicated that the single-chain PEV was more flexible than PET. In spite of the simplicity of the single-chain polymer model, the predicted *T_g_* values of PET and PEV agreed well with experimental data.

## Figures and Tables

**Table 1:** Glass transition temperature (*T_g_*) of PET and PEV predicted from our MD simulations compared to the previously reported theoretical and experimental data [10,11,27,28,29].

Polymers	Glass Transition Temperature *T_g_* (K)	
	Simulations	Experiments
PET	345, 342 [11]	350 [28], 353 [11,27]
PEV	353	347 [10], 348 [29], 356 [11]

## Data Availability

All the data generated for this publication have been included in current manuscript.

## Supplementary Materials

The following supporting information can be downloaded at: https://www.mdpi.com/article/10.3390/polym14061161/s1, Table S1: Atom names, atom types, and partial atomic charges of PET and PEV, Table S2: The assigned OPLS–AA Lennard–Jones parameters and atomic mass for PET and PEV, Table S3: The assigned OPLS–AA bond and angle parameters for PET and PEV, Table S4: The assigned OPLS–AA dihedral angle parameters for PET and PEV.
