# Supplementary material for: All-Atom Molecular Dynamics Simulations on a Single Chain of PET and PEV Polymers
Source: Polymers (Basel). 2022 Mar 14;14(6):1161. doi: 10.3390/polym14061161 (PMC8951138; doi:10.3390/polym14061161)
Supplement: Supplementary file 1 [file polymers-14-01161-s001.zip › polymers-1582649-supplementary.pdf]

polymers

MDPI

Supplementary Materials

All-Atom Molecular Dynamics Simulations on a Single Chain of PET and PEV Polymers

Mattanun Sangkhawasi [1], Tawun Remsungnen [2,*], Alisa S. Vangnai [3,4], Rungtiva P. Poo-arporn [5] and Thanyada Rungrotmongkol [3,4,6,*]

[1] Program in Biotechnology, Faculty of Science, Chulalongkorn University, Bangkok 10330, Thailand; mattajung@gmail.com
[2] Faculty of Interdisciplinary Studies, Nong Khai Campus, Khon Kaen University, Nong Khai 43000, Thailand; rtawun@kku.ac.th
[3] Center of Excellence in Biocatalyst and Sustainable Biotechnology, Faculty of Science, Chulalongkorn University, Bangkok 10330, Thailand; alisa.v@chula.ac.th (A.S.V.); thanyada.r@chula.ac.th (T.R.)
[4] Department of Biochemistry, Faculty of Science, Chulalongkorn University, Bangkok 10330, Thailand
[5] Biological Engineering Program, Faculty of Engineering, King Mongkut's University of Technology Thonburi, Bangkok 10140, Thailand; rungtiva.pal@kmutt.ac.th
[6] Program in Bioinformatics and Computational Biology, Graduate School, Chulalongkorn University, Bangkok 10330, Thailand; thanyada.r@chula.ac.th
* Correspondence: rtawun@kku.ac.th (T.R.); thanyada.r@chula.ac.th (T.R.); Tel: +66-81499-2030 (T.R.); +66 02218 05426 (T.R.)

Table S1. Atom names, atom types and partial atomic charges of PET and PEV.

	PET			PEV	
**name**	**type‡**	**charges (e)**	**name**	**type**	**charges (e)**
**Head**			**Head**		
H1	HO	0.450	H1	HO	0.450
O2	OH	−0.530	O2	OH	−0.530
C3	CO	0.635	C3	CO	0.635
O4	O	−0.440	O4	O	−0.440
C5	CA	−0.115	C5	CA	−0.115
C6	CA	−0.115	C6	CA	−0.115
C7	CA	−0.115	C7	CA	0.085
C8	CA	−0.115	C8	CA	0.085
C9	CA	−0.115	C9	CA	−0.115
C10	CA	−0.115	C10	CA	−0.115
H11	HA	0.115	H11	HA	0.115
H12	HA	0.115	O12	OS	−0.285
H13	HA	0.115	O13	OS	−0.285
H14	HA	0.115	H14	HA	0.115
C15	CO	0.625	H15	HA	0.115
O16	O	−0.430	C16	CT	0.110
O17	OES	−0.33	H17	HC	0.030
C18	CT	0.190	H18	HC	0.030
H19	HAE	0.030	H19	HC	0.030
H20	HAE	0.030	C20	CT	0.140
C21	CT	0.190	H21	HC	0.030
H22	HAE	0.030	H22	HC	0.030
H23	HAE	0.030	C23	CT	0.190
			H24	HAE	0.030
			H25	HAE	0.030

	PET			PEV	
**name**	**type‡**	**charges (e)**	**name**	**type**	**charges (e)**
**Body**			**Body**		
O24	OES	−0.330	O26	OES	−0.330
C25	CO	0.625	C27	CO	0.625
O26	O	−0.430	O28	O	−0.430
C27	CA	−0.115	C29	CA	−0.115
C28	CA	−0.115	C30	CA	−0.115
C29	CA	−0.115	C31	CA	0.085
C30	CA	−0.115	C32	CA	0.085
C31	CA	−0.115	C33	CA	−0.115
C32	CA	−0.115	C34	CA	−0.115
H33	HA	0.115	H35	HA	0.115
H34	HA	0.115	O36	OS	−0.285
H35	HA	0.115	O37	OS	−0.285
H36	HA	0.115	H38	HA	0.115
C37	CO	0.625	H39	HA	0.115
O38	O	−0.430	C40	CT	0.110
O39	OES	−0.330	H41	HC	0.030
C40	CT	0.190	H42	HC	0.030
H41	HAE	0.030	H43	HC	0.030
H42	HAE	0.030	C44	CT	0.140
C43	CT	0.190	H45	HC	0.003
H44	HAE	0.030	H46	HC	0.030
H45	HAE	0.030	C47	CT	0.190
			H48	HAE	0.030
			H49	HAE	0.030

	PET			PEV	
**name**	**type‡**	**charges (e)**	**name**	**type**	**charges (e)**
**Tail**			**Tail**		
O46	OES	−0.330	O50	OES	−0.330
C47	CO	0.625	C51	CO	0.625
O48	O	−0.430	O52	O	−0.430
C49	CA	−0.115	C53	CA	−0.115
C50	CA	−0.115	C54	CA	−0.115
C51	CA	−0.115	C55	CA	0.085
C52	CA	−0.115	C56	CA	0.085
C53	CA	−0.115	C57	CA	−0.115
C54	CA	−0.115	C58	CA	−0.115
H55	HA	0.115	H59	HA	0.115
H56	HA	0.115	O60	OS	−0.285
H57	HA	0.115	O61	OS	−0.285
H58	HA	0.115	H62	HA	0.115
C59	CO	0.625	H63	HA	0.115
O60	O	−0.430	C64	CT	0.110
O61	OES	−0.330	H65	HC	0.030
C62	CT	0.190	H66	HC	0.030
H63	HAE	0.030	H67	HC	0.030
H64	HAE	0.030	C68	CT	0.140
C65	CT	0.145	H69	HC	0.030
H66	HC	0.060	H70	HC	0.030
H67	HC	0.060	C71	CT	0.180
O68	OH	−0.566	H72	HC	0.040
H69	HO	0.300	H73	HC	0.040
			O74	OH	−0.560
			H75	HO	0.300

Table S2. The assigned OPLS-AA Lennard-Jones parameters and atomic mass for PET and PEV.

Types	σ (Å)	ε (kcal.mol^{-1})	Mass (amu)
HO	0.500	0.030	1.008
OH	3.000	0.170	15.999
O	2.960	0.210	15.999
OES	2.900	0.140	15.999
OS	2.900	0.140	15.999
CO	3.750	0.066	12.012
CA	3.550	0.070	12.012
CT	3.500	0.066	12.012
HA	2.420	0.030	1.008
HAE	2.420	0.030	1.008
HC	2.500	0.030	1.008

Table S3. The assigned OPLS-AA bond and angle parameters for PET and PEV.

	harm: $U(r)=\frac{1}{2}k_r(r-r_o)^2$			harm: $U(a)=\frac{1}{2}k_a(a-a_o)^2$	
Types	k_r (kcal/(mol·Å^2))	r_o (Å)	Types	k_a (kcal/(mol·rad^2))	a_o(°)
HO–OH	1106.000	0.945	HO–OH–CO	70.000	113.000
OH–CO	900.000	1.364	OH–CO–O	200.000	123.320
CO–O	1140.000	1.229	OH–CO–CA	170.000	111.880
CO–CA	800.000	1.490	O–CO–CA	160.000	120.400
CA–CA	938.000	1.400	CO–CA–CA	170.000	120.000
CA–HA	734.000	1.080	CA–CA–HA	70.000	120.000
CA–OS	900.000	1.364	CA–CO–OES	162.000	111.400
CO–OES	428.000	1.327	O–CO–OES	166.000	123.400
OES–CT	640.000	1.410	CO–OES–CT	166.000	116.900
OS–CT	640.000	1.410	OES–CT–HAE	70.000	109.500
CT–CT	536.000	1.529	CT–CT–HAE	75.000	110.700
CT–HAE	680.000	1.090	OES–CT–CT	100.000	109.500
CT–HC	680.000	1.090	CA–CA–OS	140.000	120.000
			CA–OS–CT	150.000	111.000
			OS–CT–HC	70.000	109.500
			HC–CT–HC	66.000	107.800

Table S4. The assigned OPLS-AA dihedral angle parameters for PET and PEV.

cos3: $U(\phi)=\frac{1}{2}[A_1(1+\cos(\phi))+A_2(1-\cos(2\phi))+A_3(1+\cos(3\phi))]$ (kcal/mol)							
Types	A_1	A_2	A_3	Types	A_1	A_2	A_3
HO–OH–CO–O	1.844	7.482	0.000	CA–OS–CT–CT	−0.657	−0.716	0.679
OH–CO–CA–CA	0.000	0.720	0.000	OS–CT–CT–HAE	0.000	0.000	0.468
CO–CA–CA–HA	0.000	7.250	0.000	OS–CT–CT–OES	2.2380	−2.327	−0.683
CO–CA–CA–CA	0.000	7.250	0.000	O–CO–OES–CT	0.000	6.000	0.000
CA–CA–CA–CA	0.000	7.250	0.000	CO–OES–CT–CT	−0.518	−0.940	0.235
CA–CA–CA–HA	0.000	7.250	0.000	CO–OES–CT–HAE	0.000	0.000	0.161
CA–CA–CO–O	0.000	2.100	0.000	OES–CT–CT–HAE	0.000	0.000	0.468
CA–CA–CO–OES	0.000	1.975	0.000	HAE–CT–CT–HAE	0.000	0.000	0.300
CA–CO–OES–CT	1.199	6.497	0.000	HC–CT–CT–OH	0.000	0.000	0.300
CA–CA–OS–CT	0.000	3.916	0.000	CT–CT–OH–HO	4.515	−3.854	0.000
CA–OS–CT–HC	0.000	0.000	0.161	HC–CT–OH–HO	0.000	0.000	0.389
